# P-931. Characterizing Sex-Specific Trends in Opioid Use Disorder-Associated Infective Endocarditis in the United States from 2016-2020

**DOI:** 10.1093/ofid/ofae631.1122

**Published:** 2025-01-29

**Authors:** D Kane Cooper, Stephen W Smith, Asher J Schranz

**Affiliations:** University of North Carolina, Carrboro, North Carolina; University of North Carolina, Carrboro, North Carolina; University of North Carolina, Carrboro, North Carolina

## Abstract

**Background:**

Injection opioid use has emerged as a leading risk factor for infective endocarditis (IE), with incidence of opioid use disorder-associated IE (O-IE) increasing nationwide. Although historically IE has predominantly impacted males, recent studied have shown drug use-associated IE (DUA-IE) may impact males and females to a similar extent. We aimed to characterize sex-specific trends and outcomes in IE across the United States (US).

**Figure:**
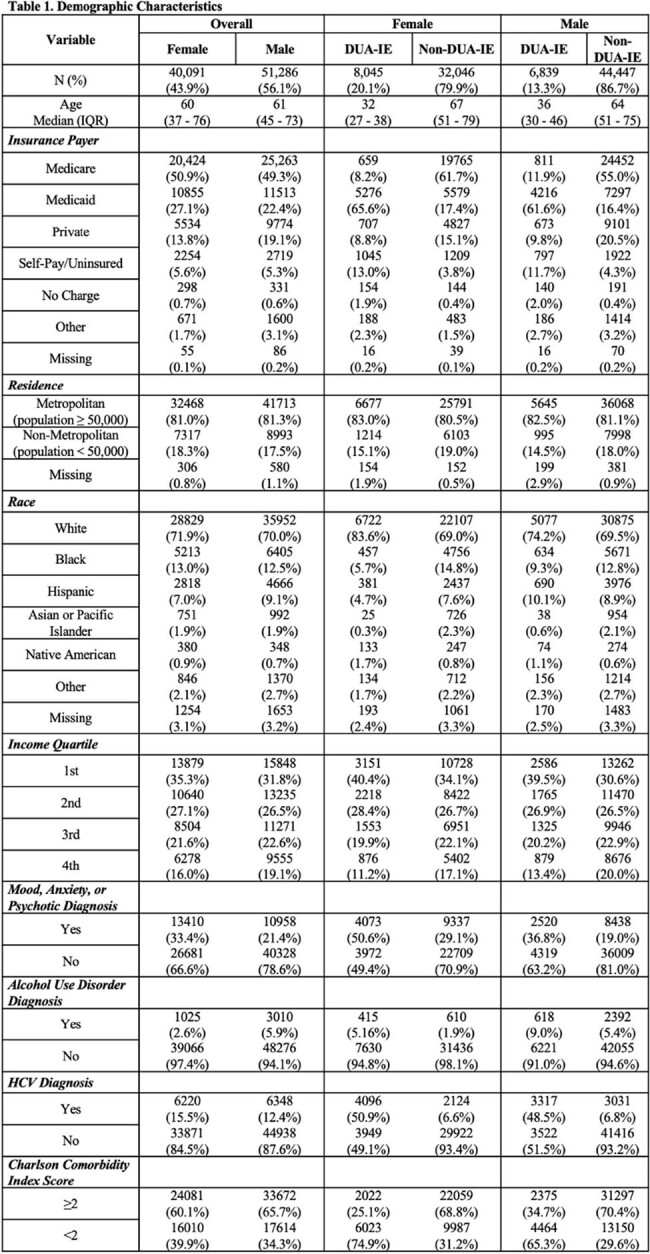

Demographic Characteristics

**Methods:**

We examined hospitalizations in the unweighted National Inpatient Sample (NIS) from 2016 to 2020. Hospitalizations coded for IE were included and stratified by sex and into O-IE and non-O-IE cohorts based on previously published ICD-10-CM codes. Demographics, clinical factors, and discharge disposition were described. Multivariable logistic regressions assessed the association between 1) sex and O-IE, and 2) sex and inpatient death, among O-IE hospitalizations.

**Figure:**
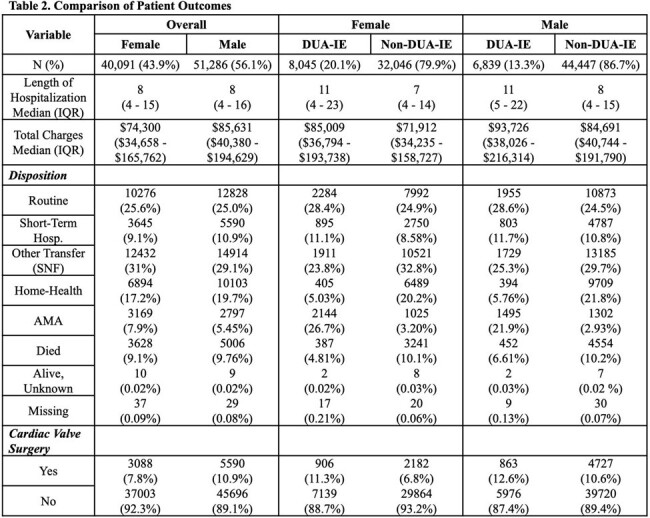

**Results:**

There were 91,377 hospitalizations for IE from 2016 to 2020 comprised of 40,091 (44%) females and 51,286 (56%) males (Table 1). Compared to 2016, hospitalizations for O-IE increased yearly for both sexes, while non-O-IE decreased (Figure 1). There were 14,884 (16%) hospitalizations due to O-IE, 54% of which were females and 46% were males. Among O-IE, females were more commonly White, insured by Medicaid and had concomitant psychiatric diagnoses. Among O-IE hospitalizations, females less commonly underwent valve surgery than males (11% vs 13%), less often died inpatient (5% vs 7%), and more frequently discharged against medical advice (DAMA) (27% vs 22%). In the adjusted models, females demonstrated 43% increased odds of O-IE diagnosis compared to males (aOR: 1.43, 95% CI: 1.32-1.54), while females diagnosed with O-IE demonstrated 21% decreased odds of inpatient mortality compared to males with O-IE (aOR: 0.79, 95% CI: 0.63-0.98).

**Figure:**
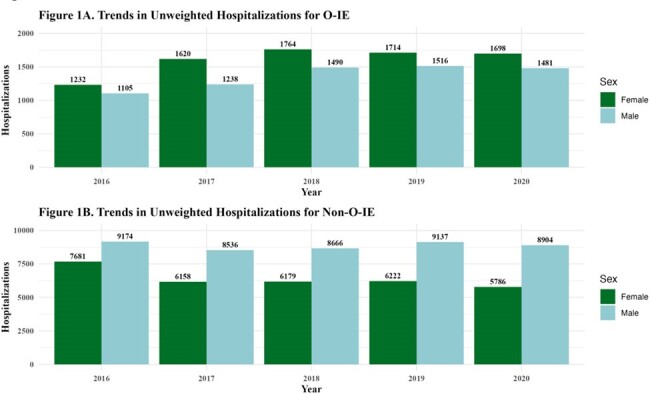

**Conclusion:**

From 2016 to 2020, most DUA-IE hospitalizations in the US were among females, in stark contrast with IE due to other causes. Females had lower odds of inpatient mortality but higher rates of DAMA. Future work is needed to characterize the social and biological factors driving sex disparities in IE hospitalizations and outcomes, which could serve as vital targets for substance use disorder and harm reduction interventions.

**Disclosures:**

**Stephen W. Smith, n/a**, Bristol Myers Squibb: Stocks/Bonds (Public Company) **Asher J. Schranz, MD, MPH**, UpToDate: I receive fees for authorship of an UpToDate article.

